# Neuregulin-1 Administration Protocols Sufficient for Stimulating Cardiac Regeneration in Young Mice Do Not Induce Somatic, Organ, or Neoplastic Growth

**DOI:** 10.1371/journal.pone.0155456

**Published:** 2016-05-13

**Authors:** Balakrishnan Ganapathy, Nikitha Nandhagopal, Brian D. Polizzotti, David Bennett, Alparslan Asan, Yijen Wu, Bernhard Kühn

**Affiliations:** 1 Department of Cardiology, Boston Children’s Hospital, Boston, MA 02115, United States of America; 2 Department of Pediatrics, University of Pittsburgh, and Richard King Mellon Institute for Pediatric Research and Division of Pediatric Cardiology, Children’s Hospital of Pittsburgh of UPMC, Pittsburgh, PA 15224, United States of America; 3 Department of Bioengineering, University of Pittsburgh, Pittsburgh, PA 15260, United States of America; 4 Department of Pediatrics, Harvard Medical School, Boston, MA 02115, United States of America; 5 Pre-clinical MRI Core, Beth Israel Deaconess Medical Center, Boston, MA 02115, United States of America; 6 Department of Developmental Biology, School of Medicine, University of Pittsburgh, Pittsburgh, PA 15224, United States of America; 7 Rangos Research Center Animal Imaging Core, Children’s Hospital of Pittsburgh of UPMC, Pittsburgh, PA 15224, United States of America; 8 McGowan Institute for Regenerative Medicine, Pittsburgh, PA 15219, United States of America; 9 Interdisciplinary Biomedical Graduate Program, University of Pittsburgh School of Medicine, Pittsburgh, PA 15261, United States of America; Emory University, UNITED STATES

## Abstract

**Background:**

We previously developed and validated a strategy for stimulating heart regeneration by administration of recombinant neuregulin (rNRG1), a growth factor, in mice. rNRG1 stimulated proliferation of heart muscle cells, cardiomyocytes, and was most effective when administration began during the neonatal period. Our results suggested the use of rNRG1 to treat pediatric patients with heart failure. However, administration in this age group may stimulate growth outside of the heart.

**Methods:**

NRG1 and ErbB receptor expression was determined by RT-PCR. rNRG1 concentrations in serum were quantified by ELISA. Mice that received protocols of recombinant neuregulin1-β1 administration (rNRG1, 100 ng/g body weight, daily subcutaneous injection for the first month of life), previously shown to induce cardiac regeneration, were examined at pre-determined intervals. Somatic growth was quantified by weighing. Organ growth was quantified by MRI and by weighing. Neoplastic growth was examined by MRI, visual inspection, and histopathological analyses. Phospho-ERK1/2 and S6 kinase were analyzed with Western blot and ELISA, respectively.

**Results:**

Lung, spleen, liver, kidney, brain, and breast gland exhibited variable expression of the NRG1 receptors ErbB2, ErbB3, ErbB4, and NRG1. Body weight and tibia length were not altered in mice receiving rNRG1. MRI showed that administration of rNRG1 did not alter the volume of the lungs, liver, kidneys, brain, or spinal cord. Administration of rNRG1 did not alter the weight of the lungs, spleen, liver, kidneys, or brain. MRI, visual inspection, and histopathological analyses showed no neoplastic growth. Follow-up for 6 months showed no alteration of somatic or organ growth. rNRG1 treatment increased the levels of phospho-ERK1/2, but not phospho-S6 kinase.

**Conclusions:**

Administration protocols of rNRG1 for stimulating cardiac regeneration in mice during the first month of life did not induce unwanted growth effects. Further studies may be required to determine whether this is the case in a corresponding human population.

## Introduction

The use of β-blockers and inhibitors of the renin-angiotensin system has significantly improved morbidity and mortality from heart failure. However, heart failure remains the leading cause of death in the United States. Despite an increase in the number of FDA applications, there have been no approvals of new medical heart failure therapeutics for more than 10 years[1]. The need for new heart failure therapies is especially high in pediatric patients because drugs developed for adults have shown no positive effects in pediatric clinical trials[1–4].

Recombinant preparations of growth factors represent a promising class of biologics for drug development. Administration of the growth factor neuregulin (NRG1) has shown benefits in adult animal models of ischemic and inflammatory heart disease[5–12]. Based on our results in neonatal mice and cultured human myocardium, we have proposed a strategy utilizing recombinant NRG1 (rNRG1) administration to stimulate cardiac regeneration in infants with heart disease[13]. Our findings indicated that a potential therapeutic window of less than 6 months of age is most likely to be effective[13].

rNRG1 preparations have investigational new drug (IND) status and are in human clinical trials (clinical trails.gov Identifier # NCT01258387, NCT01439789, NCT01214096). To date, two recombinant NRG1 preparations have been evaluated in clinical trials: a full-length peptide corresponding to amino acids 2–246[6, 12], and a neuregulin peptide corresponding to amino acids 177–237 (Nrg1β2a)[14].

The *neuregulin 1* gene is expressed in endothelial cells, glial cells in the nervous system, and stromal cells in the mammary gland[14]. The *neuregulin 1* gene product is inserted into the plasma membrane and presented to neighboring cells. Some NRG forms can be released from the cell surface by cleavage through metalloproteinases[14–16]. Surface-bound and released neuregulins interact with high-affinity cell surface receptors[17]. The neuregulin receptors ErbB2, ErbB3, and ErbB4 are receptor tyrosine kinases that belong to the EGF (epidermal growth factor)-receptor family. ErbB3 and ErbB4 are the known NRG1-binding receptor subunits. They can heterodimerize with ErbB2 to activate intracellular signaling. ErbB2 does not bind to a known ligand and its kinase domain has the potential for high activity. ErbB4 can homodimerize, and this mechanism has been proposed to activate signaling events that are different from those activated by heterodimers[18].

Germline knockouts of the *neuregulin 1*, *ErbB2*, and *ErbB4* genes in mice do not form a multi-layered myocardial wall[19–21]. The phenotype of the *ErbB3* gene knockout indicates its function in development of the endocardial cushions[22]. Multiple studies have demonstrated that *NRG1*, *ErbB2*, and *ErbB4* control cardiomyocyte cell cycle activity in mammalian and zebrafish development and regeneration [5, 8, 9, 11, 23–27]. The *ErbB2* gene is amplified in diverse types of cancer, and as a result it is a target for an effective cancer therapy using a humanized antibody, Herceptin. In summary, NRG1 and its receptors have established functions in development, cellular proliferation, and cancer[14, 17, 28–30] and represent emerging drug targets[31]. The degree to which different organs show rNRG1-stimulated effects likely depends on the pattern of receptor expression. Here, we have systematically evaluated the potential extra-cardiac growth effects of rNRG1 in mice. The present study complements our prior presentation of a therapeutic strategy for rNRG1 administration in pediatric patients[13].

## Materials and Methods

### Study design and mouse experiments

Mouse experiments were approved by Boston Children’s Hospital, Beth Israel Deaconess Medical Center (Boston, MA), and University of Pittsburgh. The study design, including the number of mice to be included and the type and time points of analyses, was predefined by the investigators for a cardiac regeneration study[13]. Because this study involved neonatal mice in which determination of gender is challenging and can be ambiguous, the design did not involve separation of results by gender. Assignment to receive treatment (rNRG1) or vehicle (BSA) was performed by B.G. Surgery was performed by BDP according to a published protocol[32]. For MRI data acquisition, investigators were blinded with the use of coded samples. MRI quantification was performed by BG, NN, DB, and YW. Study mice were not excluded based on results being outliers. Pups (ICR strain) born after 5 pm were considered day of life 0 (P0) the following day. Figs 3, 4, 5, 6, 7 and 9 show unpublished data from mice that underwent neonatal cryoinjury and were studied for a previous publication[13] (corresponding to Fig 3B and 3C).

### Administration of rNRG1

rNRG1 (R&D Biosystems, catalog # 396-HB-050/CF, corresponding to amino acids 176–246 of the human protein sequence) was dissolved in 0.1% bovine serum albumin, BSA (Sigma, catalog # A9418). Mice received daily subcutaneous injections of treatment (rNRG1, ~0.1 μg/g body weight) or vehicle (0.1% BSA) for the indicated periods. For determination of rNRG1 blood concentrations, 30–60 μL of venous blood was obtained after euthanasia. Serum rNRG1 was quantified with a proprietary ELISA protocol by a contract research organization (Intertek). The lowest reported concentration of rNRG1 in serum was 0.76 ng/mL.

### Real time polymerase chain reaction (PCR)

Mouse organs were snap frozen in liquid nitrogen immediately after resection. RNA extraction was performed with RNeasy plus Mini kit (Qiagen) according to the manufacturer’s instructions, including on-column DNase I digestion. cDNA was synthesized from 1 μg of total RNA. Eluted samples were reverse-transcribed using SuperScript III First-strand synthesis kit and oligo dT primers (Invitrogen). PCR was performed using Bio-Rad CFX384 Touch Thermal cycler and iTaq Universal SYBR Green Supermix. cDNA was amplified with the following primer pairs: Glyceraldehyde 3-phoshate dehydrogenase (GAPDH, Forward: 5’-CATCACTGCCACCCAGAAGACTG-3’, Reverse: 5’-ATGCCAGTGAGCTTCCCGTTCAG-3’), ErbB2 (Forward: 5’-GACCTCAGTGTCTTCCAGAACC-3’, Reverse: 5’-TGCGGTGAATGAGAGCCAATCC-3’), ErbB4 (Forward: 5’-ACTATATGAAGATCGCTATGCC-3’, Reverse: 5’-CCACCATTTAGTATTTCGGTCAG-3’) and NRG1 (Forward: 5’-ATCGCCCTGTTGGTGGTCGG-3’, Reverse: 5’-AGCTTCTGCCGCTGTTTCTTGGT-3’). ErbB3 primers were obtained from Bio-Rad: PrimePCR SYBR Green Assay (Assay ID qMmuCID0017615). Relative mRNA expression levels were calculated using the Livak method (2^-ΔCt^, ref. [33]).

### Body weights

During the course of rNRG1 and BSA injections, the animals were weighed once daily. Until P8, all pups of one litter were weighed together and the average weight was recorded. After P8, mice were weighed individually.

### Magnetic resonance imaging (MRI)

To quantify organ volumes, whole body MRIs were performed on day of life 35 using a Biospec 70/30 spectrometer (Bruker Biospin MRI, Billerica, MA USA) operating at 7 Tesla. Whole body MRI scans were performed on day of life 191 using a Biospec 94/20 spectrometer (Bruker Biospin MRI, Billerica, MA USA) operating at 9.4 Tesla. Both instruments were equipped with an actively shielded gradient system and a quadrature radio-frequency volume coil with an inner diameter of 72 mm. Anesthesia was induced with oxygen and isoflurane mixture (3% isoflurane) and maintained with 1.5% isoflurane applied via facemask. The animal’s respiration was monitored during the entire imaging procedure. The body temperature of the mice was maintained at approximately 37°C via circulation of warm water in the animal bed. To generate whole-body magnetic resonance images a 3D RARE pulse sequence was used with the following parameters: repetition time (TR) = 400 ms, effective echo time (TE) = 23.72 ms, echo train length = 8, number of averages (NEX) = 2, acquisition matrix = 400 x 128 x 140 or 400 x 128 x 128, field-of-view (FOV) = 80 mm x 36 mm x 28 mm or 80 mm x 36 mm x 25.6 mm yielding a voxel resolution of 0.20 mm x 0.28 mm x 0.20 mm.

In addition to whole-body MRI, higher resolution MRI for abdomen and brain were acquired with the following parameters: repetition time (TR) = 1 sec, effective echo time (TE) = 12 ms, RARE factor = 8, effective TE = 48 ms, number of averages (NEX) = 2, acquisition matrix = 512 x 512, field-of-view (FOV) = 30 mm x 30 mm yielding a voxel resolution of 59 μm x 59 μm for the abdomen; repetition time (TR) = 2141 ms, effective echo time (TE) = 12 ms, RARE factor = 8, Effective TE = 48 ms, acquisition matrix = 512 x 512, field-of-view (FOV) = 25 mm x 25 mm yielding a voxel resolution of 49 μm x 49 μm were used for the brain. Researchers who analyzed whole-body MRI scans for tumor growth and organ size were unaware of the assignment to treatment and control.

### Analysis of MRI scans for mammary gland tumor growth

The MRI scans were examined for masses embedded in the mammary fat pads along both the lateral sides of the lower chest and abdomen in the 3D whole body scans and the lateral part of the chest from cardiac cine MRI images[34, 35].

### Necropsy, organ weights, and resection

Mice were anesthetized with 1–3% of isoflurane in oxygen. Body cavities were opened, inspected for tumors, and photographs were taken. All organs were washed in cardioplegia solution (50 mM KCl/PBS) and dabbed with a gauze pad and then weighed. Briefly, organs were weighed in the following ways. Heart: the blood was expressed from the cavities before weighing. The body weight was measured after resecting the heart, but before other organs were removed. Paired organs were weighed together. The left tibia length was measured with an office ruler.

### Western blot

Organs were lysed in RIPA buffer (Pierce Thermo Scientific, Rockford, IL) containing 1X Halt protease inhibitor (Thermo Scientific, Rockford, IL) followed by incubation on ice for 5 min. The samples were sonicated (Fisher Scientific, 50% pulse, 30 sec, 4°C). The lysate was then incubated for 15 min on ice, followed by centrifugation at 14,000*g*. The supernatant was aliquoted to 1.5 mL Eppendorf tubes and flash frozen. Bicinchoninic acid (BCA, Pierce Thermo Scientific, IL) assay was performed to quantify protein concentrations. Thirty μg of protein was loaded onto 4–20% pre-cast gradient SDS-PAGE gels (Bio-Rad, Berkeley, CA catalog # 4561093) and further analyzed by Western blotting. The bands were transferred to a nitrocellulose membrane (Bio-Rad, Berkeley, CA, catalog #1704158) using a Trans-Blot Turbo transfer system (Bio-Rad, Berkeley, CA). The blots were probed with phospho-ERK1/2 (Cell Signaling, catalog #4370) or total ERK1/2 (Cell Signaling, catalog #4695) primary antibodies and anti-rabbit IgG horseradish peroxidase linked secondary antibodies. Antibodies were used at a 1:1,000 dilution. The blots were developed using ECL Western blotting substrate (Pierce Thermo Scientific, Rockford, IL) and imaged using the ChemiDoc MP imaging system (Bio-Rad, Berkeley, CA). The phospho ERK1/2 abundance levels were quantified using Image J and normalized to their corresponding total ERK1/2 abundance.

### ELISA

Ten μg of organ lysate prepared as described above was loaded onto PathScan Phospho-p70 S6 kinase plates (Cell Signaling, catalog #7063C) or PathScan Total p70 S6 kinase plates (Cell Signaling, catalog #7038C) and the assay performed according to manufacturer’s instructions.

### Histopathological analyses

Organs such as lungs, spleen, liver, kidney and brain of animals from different treatment groups were harvested and fixed in 3.7% formaldehyde overnight at room temperature. The organs were washed in 1X phosphate buffered saline (PBS), transferred to 30% (wt/vol) sucrose solution in PBS and incubated overnight at 4°C. Organs were embedded in OCT tissue freezing media, sectioned (10–14 μm), and subjected to Hematoxylin and Eosin staining (H&E, Sigma Aldrich) following manufacturer’s instructions. Photomicrographs were taken using a Nikon 90i microscope with a color camera (Photometrics). One section per organ was examined.

### Statistical analyses

Numerical results are represented as means ± SEM. Continuous outcomes were compared with analysis of variance (ANOVA) followed by Bonferroni’s post hoc testing. Statistical significance was achieved with a two-sided P value < 0.05. Statistical analyses were performed with GraphPad Prism, version 6.

## Results

### Genes for the NRG1 receptors and *NRG1* are broadly expressed

**Fig 1:**
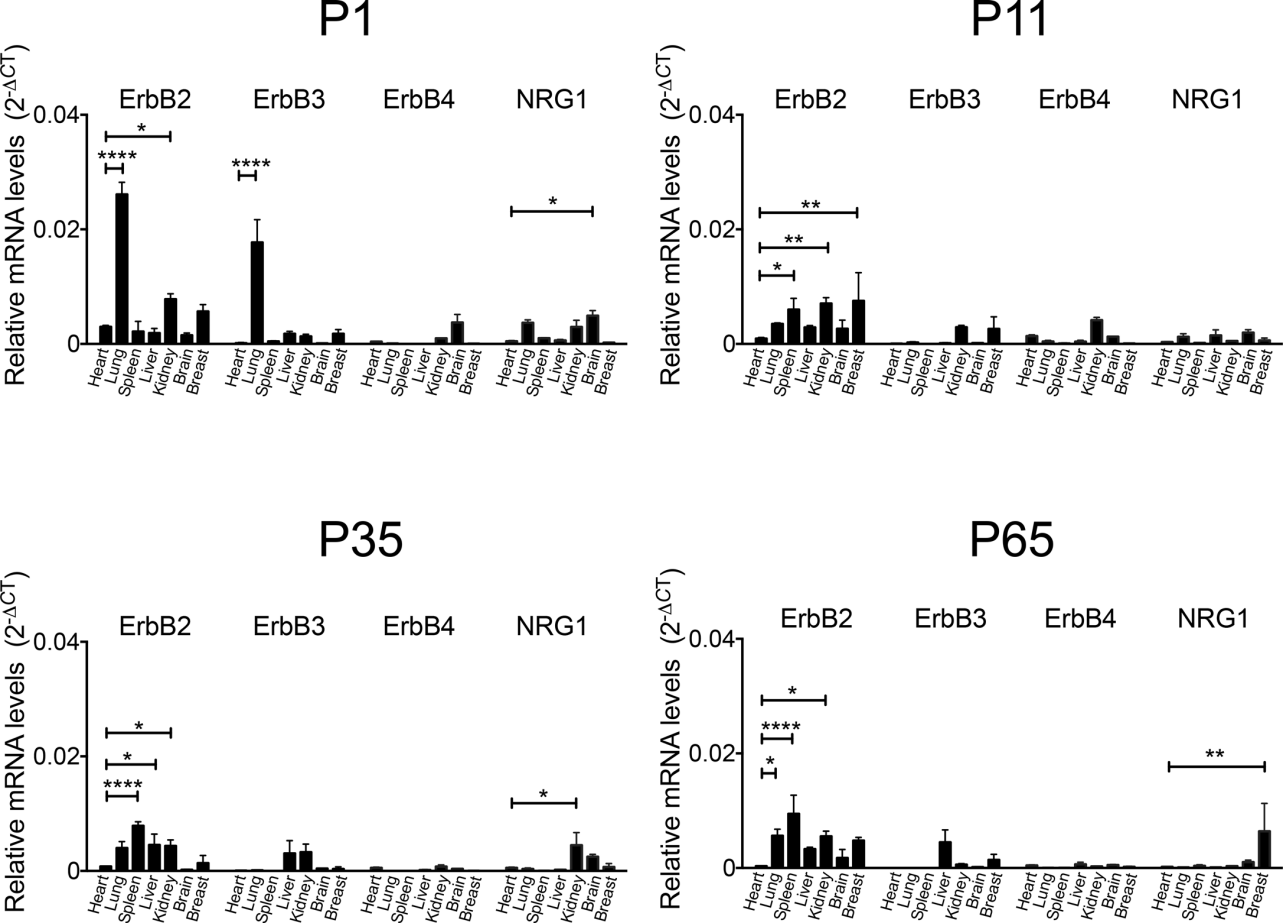
ErbB receptors are broadly expressed. Mice were euthanized on day of life 1, 11, 35, and 65. Real time PCR showed all examined organs expressed ErbB receptor subunits. Statistical significance was tested in comparison to heart samples for each time point by analysis of variance (ANOVA) followed by Bonferroni’s multiple comparison test. *P<0.05, **P<0.01, ***P<0.001, ****P<0.0001.

NRG1 receptor expression was reported in many adult organs (reviewed in [14]) and in some types of cancer. To systematically and consistently determine the expression patterns of NRG1 and its receptors in the period under investigation, we examined multiple tissues on day of life 1, 11, 35, and 65 with real time PCR (RT-PCR, Fig 1, n = 3 biological replicates, each run in triplicates). Data were normalized to corresponding GAPDH levels, graphed, and compared with expression levels in the heart (Fig 1). The level of expression in the heart was included in the analyses as a positive control. *ErbB2* receptors were expressed in all tissues analyzed. *ErbB3* receptors were not expressed in the heart at any time point. *ErbB4* receptor expression did not vary between tissues and time points. This indicates that the ErbB2/ErbB4 heterodimer is the prevalent neuregulin receptor configuration in the heart. *ErbB2* expression in the heart decreased from P1 to P10 mice, consistent with recently published data[11]. NRG1 receptor expression was significantly higher in the brain compared to the heart in P65 mice (*P*<0.01). In summary, all examined organs expressed ErbB genes at one or more of the examined time points. In conclusion, all of the examined organs have the potential to form high-affinity neuregulin receptors and thus have the potential to respond to rNRG1-administration.

### A single, weight-adjusted injection of rNRG1 induces transient increases of serum concentrations

**Fig 2:**
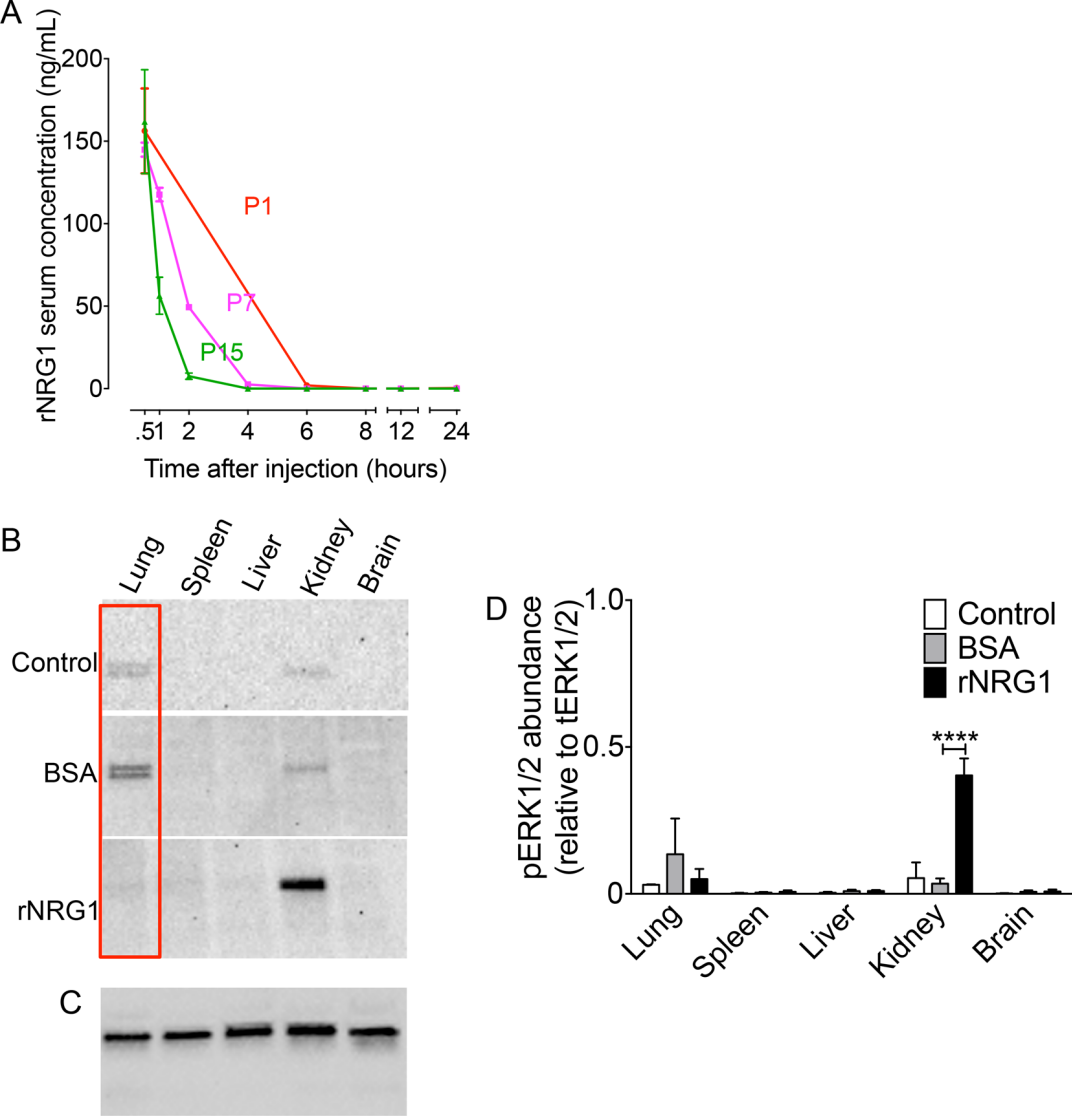
A single weight-adjusted rNRG1-injection in neonatal mice induces similar peak rNRG1 concentrations in serum and significant increases in phospho ERK1/2 in kidney. Mice of indicated ages received one subcutaneous injection of rNRG1 (0.1 μg/g). (A) The serum concentration of rNRG1 was determined by ELISA. (B-D) Mice either received no injection (control) or one BSA or rNRG1 injection on day of life 7 (P7) and organs were resected 1.5 hours later. Western blots showed phospho-ERK1/2 (B), and total ERK1/2 levels (C) in organs analyzed. Red rectangles indicate an example of corresponding control and treatment groups (B). Total ERK1/2 loading control is a representative example of all treatment groups (C). Quantification of phospho-ERK1/2 abundance normalized to their respective total ERK1/2 loading controls (D). Statistical analysis was tested with ANOVA followed by Bonferroni’s multiple comparison test (D). ****P<0.0001. Error bars indicate SEM. *n* = 2 for P1, *n* = 3 for P7, and P15 (A); *n* = 3 (B-D).

Because pharmacokinetics may differ between neonatal and adult mice, we determined the serum concentrations after a single, weight-adjusted subcutaneous injection of rNRG1 (100 ng/g) at different ages (day of life 1, 7, and 15). This dose was previously shown to stimulate cardiomyocyte proliferation in mice[5, 13] and is similar to the concentration of rNRG1 used to stimulate cycling of primary cardiomyocytes in culture (100 ng/mL cell culture media)[5, 24]. However, this dose is significantly higher than doses that are currently applied in humans (0.6–2.5 ng/g body weight[6, 12]). At 30 min after injection of 100 ng/g body weight, the peak serum concentrations were 150–200 ng/mL in all age groups tested (Fig 2A). These peak serum concentrations are within the range of the concentrations of endogenous NRG1 measured in humans[36, 37]. Blood rNRG1 levels at 4 hr indicated that in P7 and P15 mice, rNRG1 concentrations were near baseline. Blood rNRG1 levels at 6 hr indicated that in P1 mice, levels were near baseline. Thus, the time until rNRG1 was cleared from the serum was equal to or less than 4 hr in P7 and P15 mice and equal to or less than 6 hr in P1 mice (Fig 2A). In conclusion, a single weight-adjusted dose of rNRG1 induces transient increases of serum concentration in mice.

### A single dose of rNRG1 increases phospho ERK1/2 in the kidney

Since the detected *ErbB* and *NRG1* mRNAs indicated variable expression in different organs, it was of interest to determine if a single dose of rNRG1 administration activated intracellular signaling pathways. Extracellular receptor regulated kinase (ERK1/2) is the primary pathway activated by ErbB receptors [28, 38]. Accordingly, we examined the changes in phospho ERK1/2 abundance using Western blots (Fig 2B). The phospho-ERK1/2 abundances were normalized to their corresponding total ERK1/2 (Fig 2C). A 1,065% increase in phospho-ERK1/2 abundance was detected in the kidneys of rNRG1 treated mice (1.5 hours after injection) on day of life 7 (Fig 2D). None of the other organs exhibited a significant increase in phospho ERK1/2. In conclusion, our rNRG1 administration protocol activated cellular pathways outside of the heart.

### Design of pre-clinical trials of rNRG1 administration in young mice

**Fig 3:**
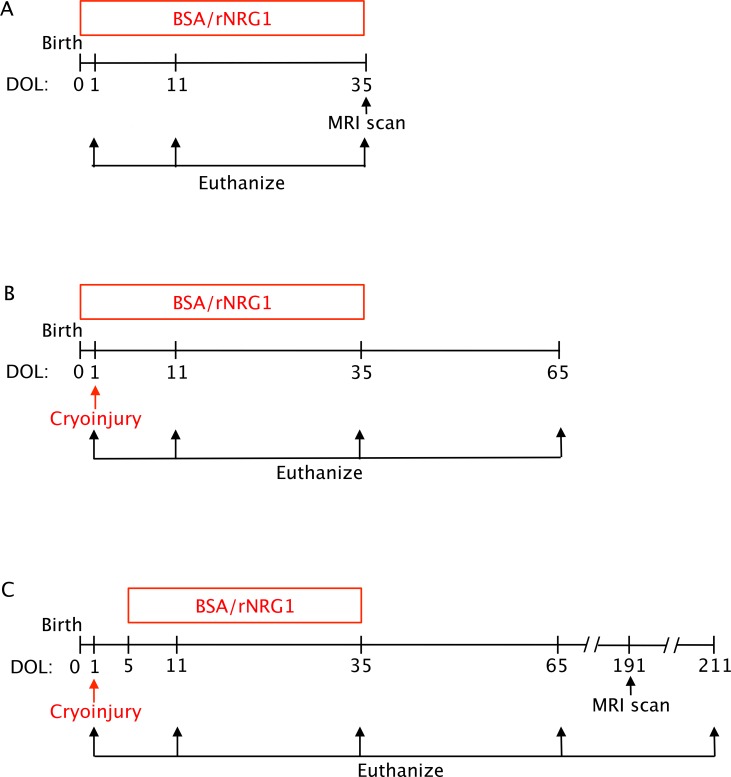
Diagrams of study design. Daily injections of vehicle (BSA) and rNRG1 were begun on the day of life 0 (A, B) and day of life 5 (C) and are indicated with red boxes. Cryoinjury was performed on day of life 1 (P1, B, C). DOL, day of life.

To detect unwanted growth effects induced by rNRG1, we examined data from mice without heart injury and cryo-injured mice that had received BSA or rNRG1 in the first month of life for the stimulation of cardiac regeneration[13]. rNRG1 administration began on the day of birth in mice without heart injury (Fig 3A) and with cardiac cryoinjury (Fig 3B). In a third, long-term study, rNRG1 administration began on day of life 5 in mice with cardiac cryoinjury (Fig 3C). The *in vivo* part of each study was performed according to a pre-designed protocol. We considered growth during the time of rNRG1-administration as primary outcome and growth until 6 months after rNRG1-administration as secondary outcomes.

### Administration of rNRG1 does not alter somatic growth in mice

**Fig 4:**
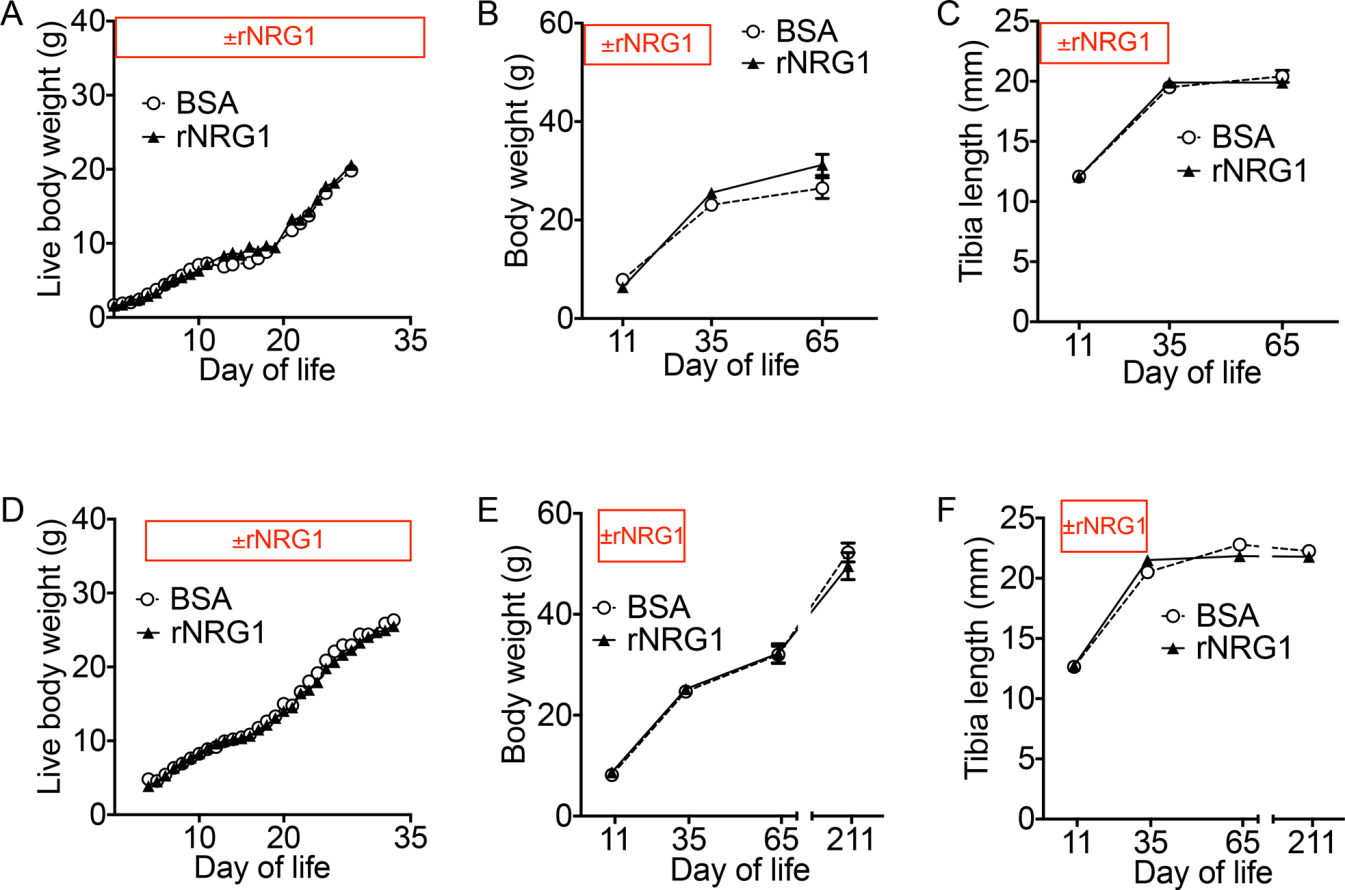
Administration of rNRG1 in young mice does not alter somatic growth. Mice underwent cryoinjury on day of life 1 (P1) and received daily BSA or rNRG1 injections (indicated by ± rNRG1) between day of life 0 and 35 (early, A-C) and day of life 5 and 35 (late, D-F). Live body weights (A, D), carcass body weights after resection of heart (B, E), and tibia length (C, F) show no significant difference (*p*>0.05) between BSA and rNRG1 treated mice. Statistical analysis by analysis of variance (ANOVA) followed by Bonferroni’s multiple comparison test.

We analyzed the body weights to determine a potential effect on body growth. The live body weight was not different between BSA and rNRG1-treated mice during the period of administration (Fig 4A and 4D). The body weight after resection of internal organs was not different between BSA and rNRG1-treated mice at 10, 34, and 64 dpi (Fig 4B and 4E). The tibia length increased until 34 dpi and was not different between BSA and rNRG1-treated mice (Fig 4C and 4F), indicating that the period of rNRG1 administration covered the entire skeletal growth phase in mice. We followed a group of mice until they were 7 months of age to determine potential late effects (Fig 4E and 4F). At the age of 7 months, *i*.*e*. 6 months after cessation of rNRG1 administration, body weight and tibia length were not different. In conclusion, rNRG1-administration did not alter skeletal growth or growth of body mass.

### Administration of rNRG1 does not alter adult organ volumes

**Fig 5:**
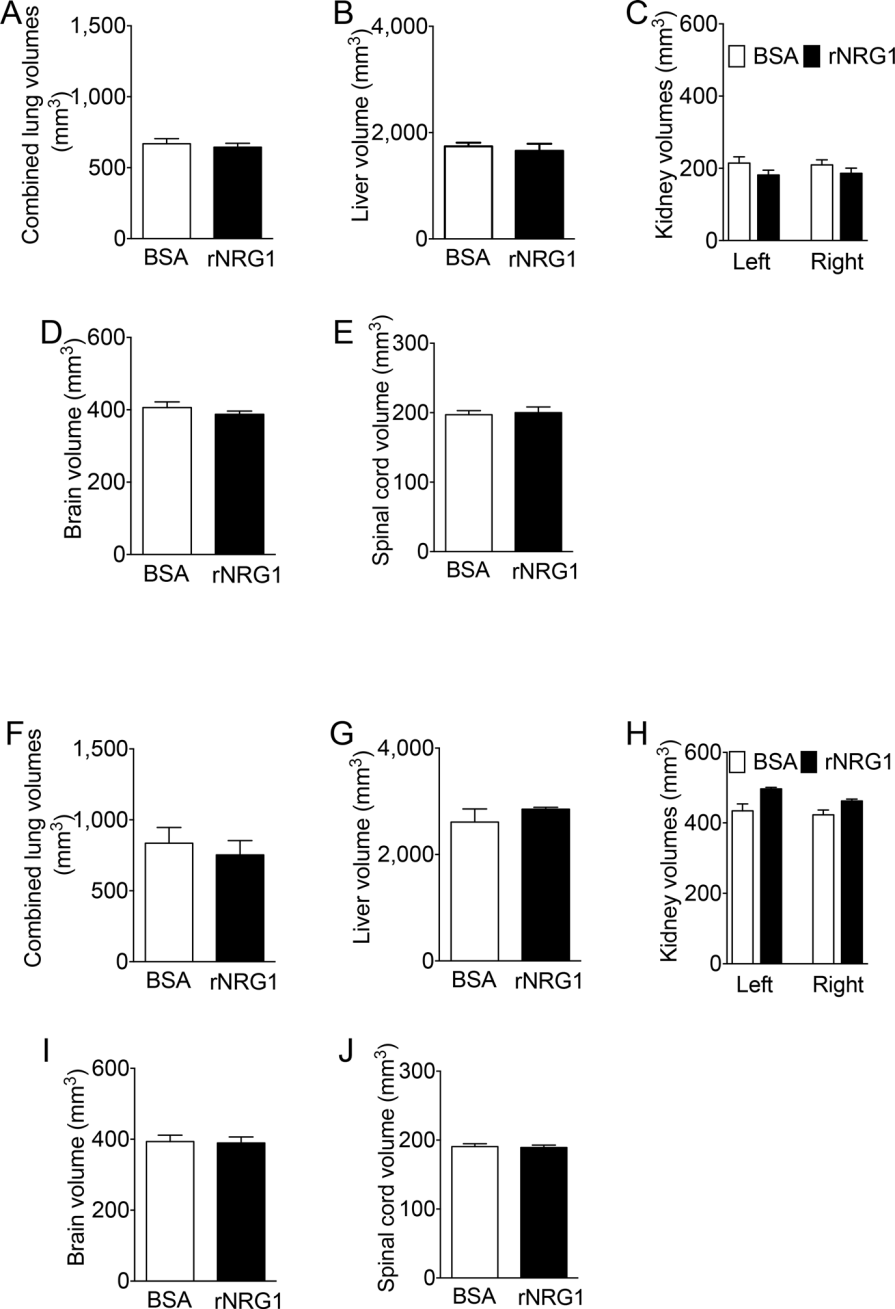
Administration of rNRG1 has no effect on adult organ volumes. Mice received daily BSA or rNRG1 injections between day of life 0 and 35 without heart injury (A-E), and day of life 5 and 35 and received cryoinjury on day of life 1(F-J). Lung (A, F), liver (B, G), kidney (C, H), brain (D, I), and spinal cord (E, J) volumes were determined by MRI and were not statistically different (*p*>0.05) between BSA and rNRG1 treated mice. Statistical analysis by Student’s *t* test (A, B, D-G, I, J) and analysis of variance (ANOVA) followed by Bonferroni’s multiple comparison test (C, H).

**Fig 6:**
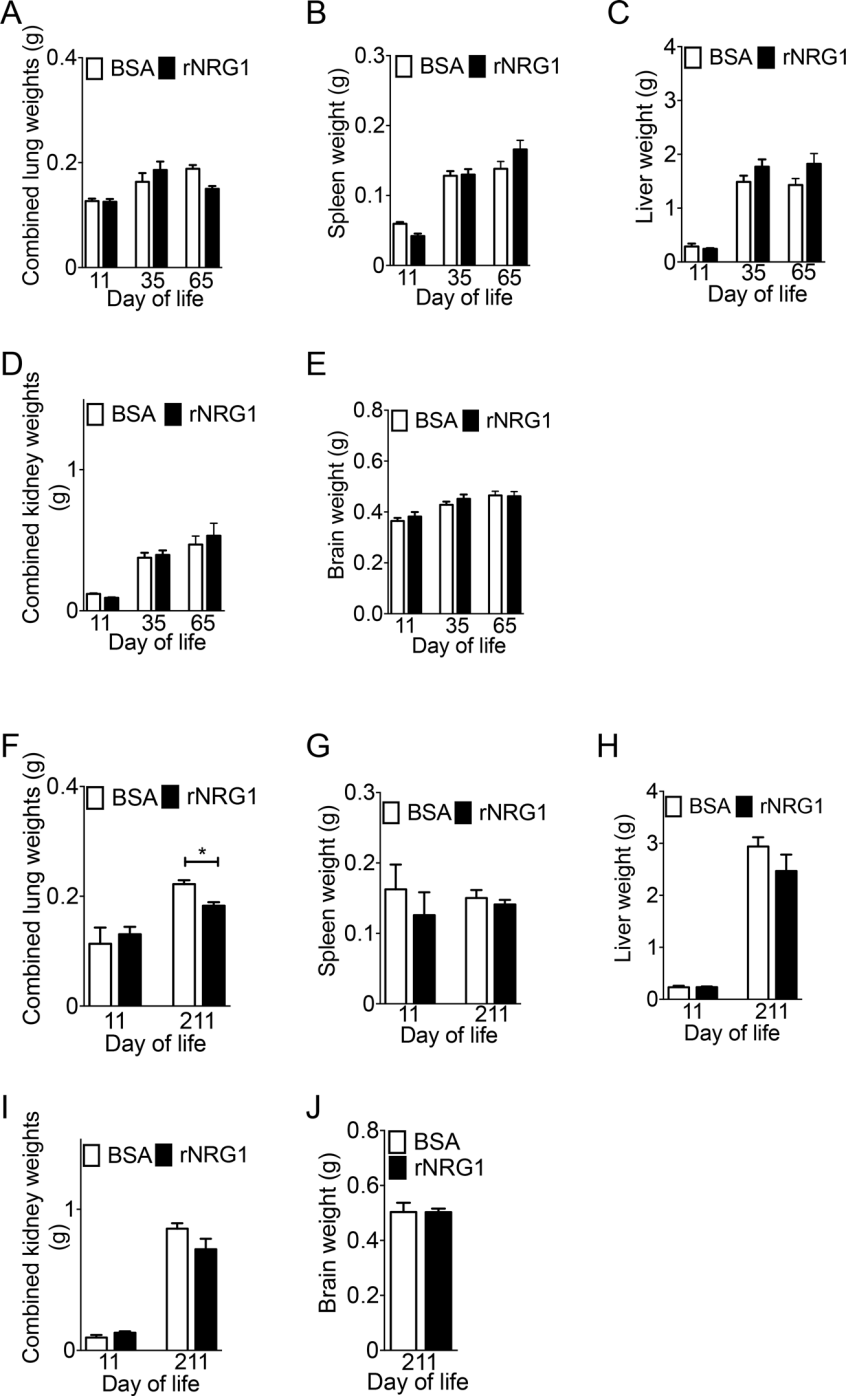
Administration of rNRG1 in neonatal mice does not alter organ growth. Mice underwent cryoinjury on day of life 1 (P1) and received daily BSA or rNRG1 injections between day of life 0 and 35 (early, A-E) and day of life 5 and 35 (late, F-J). (A, F) Lung weights of rNRG1 mice were significantly lower at day of life 211 in late administration (F). (B-E, G-J) Spleen (B, G), liver (C, H), kidney (D, I), and brain (E, J) weights were not significantly different. Statistical analysis by Student’s *t* test (J) and analysis of variance (ANOVA) followed by Bonferroni’s multiple comparison test (A-I). *P<0.05.

It is of interest to determine whether administration of rNRG1 during the somatic growth phase affected the growth of internal organs. To measure organ volume, we performed whole-body MRI scans. The volumes of lungs, liver, kidney, brain, or spinal cord were not significantly different between the BSA and rNRG1 treated mice immediately (Fig 5A–5E) and 6 months (Fig 5F–5J) after the cessation of rNRG1-administration.

### Administration of rNRG1 has no effect on organ weights

We compared the organ weights between BSA and rNRG1-treated mice (Fig 6). We noted that lung weights decreased by 18% in rNRG1 treated mice at day of life 211 (*P*<0.05, ANOVA, Fig 6F). In this context, it is important to note that although the lung weights in rNRG1 mice were significantly decreased, the lung volumes were not (Fig 5A and 5F). There was no difference in combined lung weights in the early administration. The spleen, liver, kidney, and brain weights were not different between the BSA and rNRG1 treated mice in both early and late administration (Fig 6B–6E and 6G–6J).

### Administration of rNRG1 does not induce neoplastic growth

**Fig 7:**
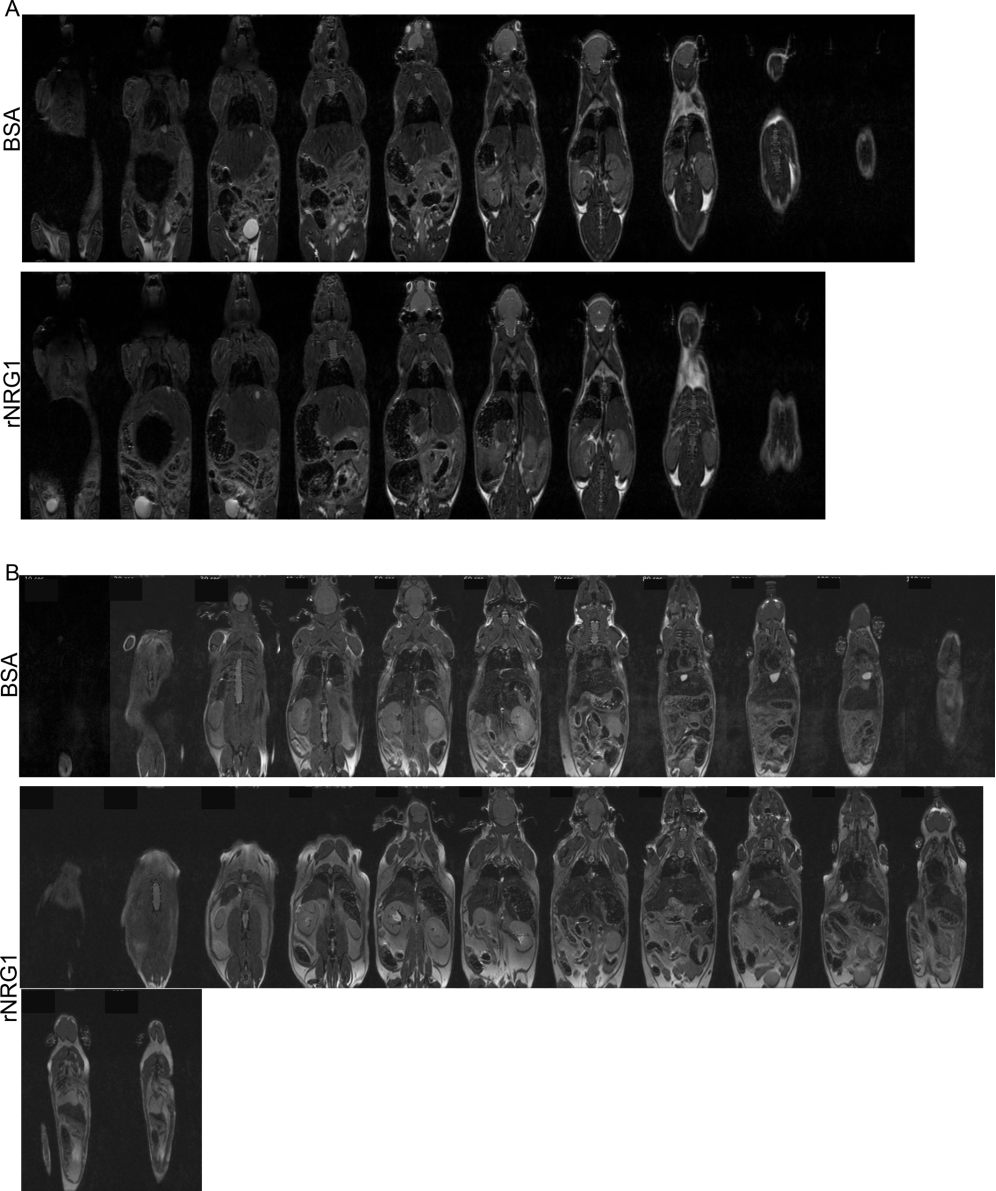
Administration of rNRG1 during the somatic growth phase does not induce neoplastic growth at the organ level. Mice received daily BSA or rNRG1 injections between day of life 0 and 35 (A), and, day of life 5 and 35 (B). Cryoinjury was performed on day of life 1 (B). (A-B) Representative whole body MRI scans for a BSA and rNRG1-treated mice on day of life 35 (A) and day of life 191 (B).

**Fig 8:**
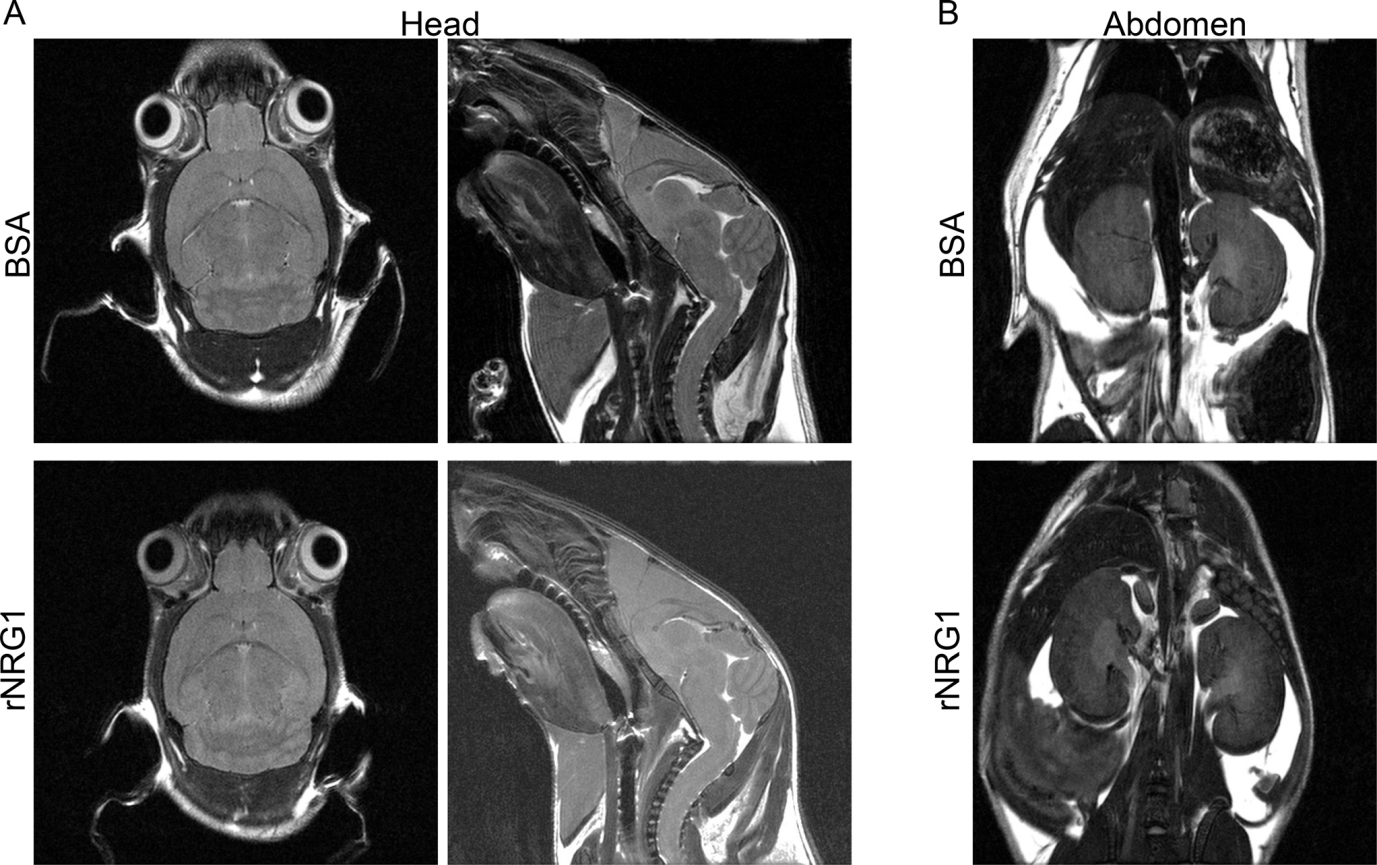
Administration of rNRG1 during the somatic growth phase does not induce neoplastic growth in the head and abdomen. Mice received daily BSA or rNRG1 injections between day of life 0 and 35. Representative examples of sagittal (A, left panels), coronal (A, right panels) MRI scans of brain, and abdomen (B) for a BSA and rNRG1-treated mice on day of life 35 are shown.

*ErbB2* gene amplification in cancer causes constitutive signaling. Although the molecular mechanisms activated by rNRG1-administration are different from constitutive receptor activation, it is important to determine an effect on neoplastic growth. We performed whole body MRI imaging studies at 35 days (Fig 7A, n = 6) and 191 days (Fig 7B, n = 3) of age. High-resolution MRI of the head and abdomen was performed at 35 days of life (Fig 8A and 8B). No visible tumor growth or mass was found in the organs, mammary fat pads, lateral chest walls, spinal cord, abdomen, and the surrounding tissues in any of the rNRG1 treated groups. Inspecting the open body cavities and exposed organs at day of life 11 (n = 17), 35 (n = 17), 65 (n = 11), and 211 (n = 7) showed no visible tumors in the rNRG1 treated mice. In conclusion, rNRG1 administration did not induce tumor formation in mice.

### Administration of rNRG1 does not induce neoplastic foci formation

**Fig 9:**
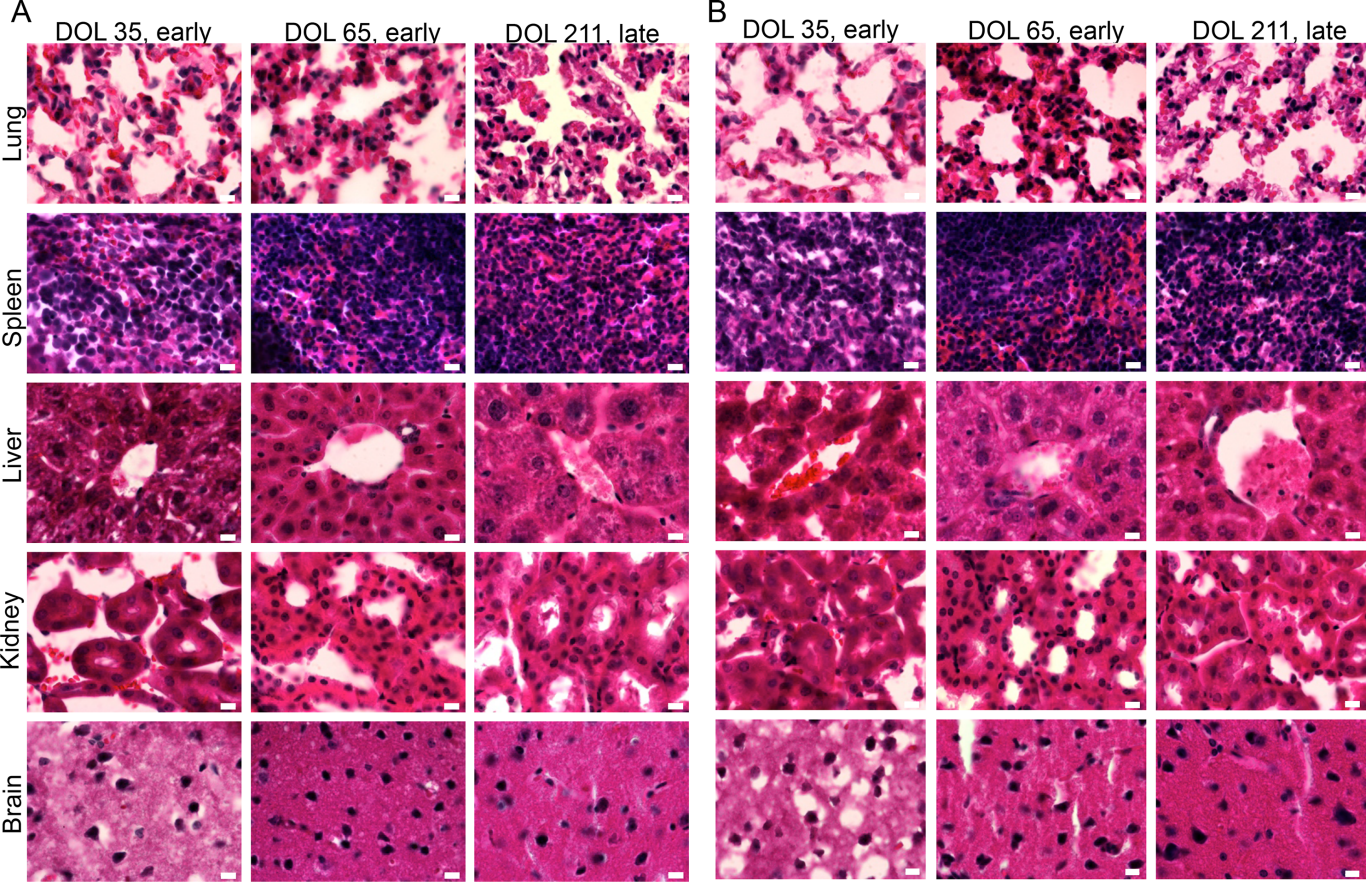
Administration of rNRG1 during the somatic growth phase does not induce the formation of neoplastic foci. Mice received daily BSA (A) or rNRG1 (B) injections between day of life 0 and 35, and organs were resected at the indicated time points. Cryoinjury was performed on day of life 1 (A, B, middle and right panels). Organs were sectioned and subjected to Hematoxylin and Eosin (H&E) staining. Representative images from each organ are shown. Scale bar, 10 μm. DOL, day of life.

While we did not find evidence for neoplastic tumor growth, we examined the organs for early stage neoplastic foci with microscopy. We did not find evidence for microscopic neoplastic foci in BSA or rNRG1-treated groups (Fig 9).

### Daily administration of rNRG1 has no effect on phospho ERK1/2

**Fig 10:**
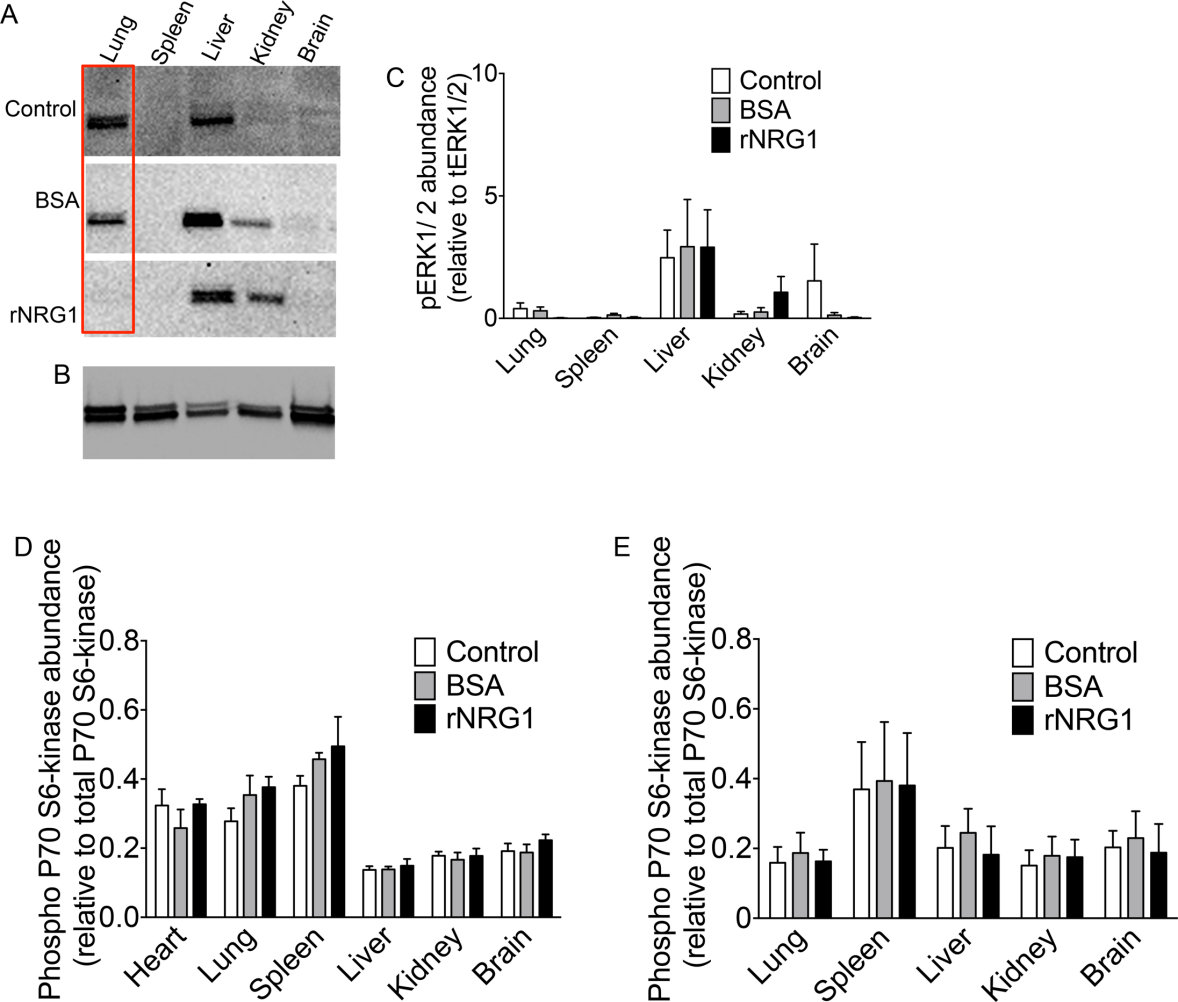
Daily administration of rNRG1 does not increase phospho ERK1/2 and S6 kinase levels. Mice received no injections (control) or daily BSA or rNRG1 injections between day of life 0 and 35 (A-D) and organs were resected on day of life 35 (24 hours after the last injection). In another experiment, mice received no injection (control) or a single dose of BSA or rNRG1 injection on day of life 7 and organs were resected 1.5 hours later (E). (A, B) Western blots showed no significant difference in phospho-ERK1/2 abundance between control and treatment groups (A), and total ERK1/2 levels (B). Red rectangles indicate an example of corresponding lanes in control and treatment groups (A). Total ERK1/2 loading control is a representative example of all treatment groups (B). (C) Quantification of phospho-ERK1/2 abundance normalized to their corresponding total ERK1/2 levels. (D, E) Quantification of phospho P70-S6 kinase abundance by ELISA normalized to corresponding total P70-S6 kinase levels were unaltered on day of life 35 (D) and day of life 7 (E). Statistical analysis was tested with ANOVA followed by Bonferroni’s multiple comparison test (C-E).

We examined the phosphorylation levels of ERK1/2 in organs after 35 daily injections to determine the effects of long-term rNRG1 administration and found no significant difference in BSA or rNRG1-treated groups (Fig 10A–10C).

### Daily administration of rNRG1 has no effect on S6 kinase activity

Since administration of rNRG1 was not associated with organ growth, despite increased pERK1/2 (Fig 2B–2D), we examined the pERK1/2 activation pattern after multiple daily injections. Mice that received 35 days of daily rNRG1 administration showed a non-significant increase of phospho-ERK1/2 in kidney (Fig 10A–10C). Activation of ERK1/2 is connected to phosphorylation of S6 kinase[38]. Because S6 kinase regulates cellular growth and proliferation, we compared its activation between BSA and rNRG1-treated mice. Phospho-p70 S6 kinase was not increased by rNRG1 administration (Fig 10D). This indicates that daily rNRG1 administration for 35 days did not stimulate mechanisms of cellular growth or proliferation. This prompted us to re-examine the protein lysates of organs taken 1.5 hours after a single injection of rNRG1 in P7 mice. Phospho-S6 kinase was not increased by rNRG1 (Fig 10E). In conclusion, administration of rNRG1, shown to be bioactive by pERK1/2 increase in the kidney (Fig 2B and 2D), does not activate mechanisms of cellular growth or proliferation. This is consistent with the lack of increased organ growth in rNRG1-treated mice.

## Discussion

In this study, we focused on the analysis of growth effects of rNRG1 administration outside of the heart. The presented results demonstrate that administration of rNRG1 protocols sufficient for stimulating cardiomyocyte cycling and cardiac regeneration does not induce somatic, organ, or neoplastic growth in mice. The dose of rNRG1 administered (which we have previously shown to be sufficient for stimulating cardiomyocyte cycling and cardiac regeneration[13]) induced a significant rise in the concentration of circulating rNRG1 in serum in the current study. The dose and frequency of rNRG1 administered in our study are significantly higher than in reported human phase I trials (0.6–2.5 ng/g body weight[6, 12]). Yet, we did not detect rNRG1-induced somatic, organ, or neoplastic growth, or early stage neoplastic foci. This finding is important in the context of ongoing efforts to develop rNRG1 into a biological therapeutic for humans with heart failure. As such, our findings deserve further discussion.

The peak rNRG1 concentrations that we measured in serum are within the range of the concentrations of endogenous NRG1 measured in humans[36, 37]. This offers one possible explanation for the lack of general growth effects. Mechanistically, the absence of rNRG1-induced organ growth can be explained with the lack of increased S6 kinase phosphorylation (Fig 10D and 10E). This finding indicates that rNRG1 administration does not activate cellular mechanisms of protein synthesis and can explain the lack of increased organ weight.

Considering the cellular targets of rNRG1 could provide additional explanations for the lack of unwanted growth effects. All organs that were tested in this report showed expression of NRG1-receptors (Fig 1)[14]. The expression pattern of messenger RNA for *ErbB* receptor subunits (Fig 1) is consistent with the presence of NRG1-binding sites as determined with radioligand binding[39]. To explain the lack of organ growth in the presence of receptor expression, one has to consider that the effects of rNRG1 *via* activation of the receptor are controlled by desensitization mechanisms. These mechanisms involve down-regulation of the receptor and intracellular signal transduction pathways, leading to protection against over-stimulation of cellular responses. In addition, our regimen of once-daily rNRG1-administration induced transient increases in serum, which are unlikely to induce constitutive receptor activation. In contrast, the biochemical mechanisms leading to over-activation of cellular proliferation in cancer are fundamentally different. In cancer, amplification of the *ErbB2* gene, encoding the subunit of the heterodimeric *NRG1*-receptor with high kinase activity induces constitutively high protein levels. High levels of receptor expression induce ligand-independent activation[28] which is mechanistically different from ligand-induced activation.

It is theoretically possible that rNRG1 could induce escape of a small sub-population of cells into uncontrolled or neoplastic proliferation, which may initially be below the detection threshold of MRI and visual inspection. To address this possibility, we carried out a follow-up assessment at 210 days postnatally, which should have allowed sufficient time for such uncontrolled proliferation to lead to visible or measurable organ or tumor growth. The results show that this was not the case. In addition, we have examined young mice that received 35 daily injections of rNRG1 with both low-resolution (0.2mm x 0.28mm x 0.2mm) whole-body MRI as well as high-resolution (up to 49 μm in-plane resolution) MRI to cover the whole volume of the body. In all cases examined, no disruption of the normal anatomy due to neoplastic growth was detected. Thus, it is unlikely that rNRG1 treatment induced neoplastic growth larger than 49 μm (Fig 8A and 8B). These MRI finding are corroborated by the absence of microscopic neoplastic foci (Fig 9A and 9B).

It should be noted that constitutive expression of NRG1 (glial growth factor β3) in Schwann cells induces peripheral nerve sheath tumors[40]. Early passages of these tumors showed increased Ras activation, compared with non-neoplastic Schwann cells, suggesting activation of MAP-kinase/ ERK pathway. Our findings appear to disagree with the results of Kazmi *et al*[40] on 2 points: We did not find evidence for nerve sheath tumor formation nor activation of the ERK1/2 pathway in the nervous system (Figs 2B–2D and 10A–10C). These differences can be explained by the transient nature of biological activation induced by injections of rNRG1 (Fig 2A).

Another example of apparently discrepant results is the reported activation of spermatogonia proliferation in organ cultures of testicular fragments from newt by newt NRG1[41]. However, *in vivo* spermatogonia and male germ cells are protected by the blood-testis barrier, which is impermeable for macromolecules such as rNRG1[41, 42].

In summary, this preliminary study characterizing biosafety of rNRG1 administration showed no induction of extra-cardiac growth effects in young mice. Further animal and human studies are required to establish a comprehensive safety profile.
